# Exogenous fungal endophthalmitis in a potato farm worker

**DOI:** 10.4102/sajid.v36i1.329

**Published:** 2021-12-17

**Authors:** Ghowa Booley, Raphael Chanda, Priscilla Daries, Clive Misland, Adrian J. Brink, Leandri Linde, Christoffel J. Opperman

**Affiliations:** 1 Department of Pathology, Faculty of Health Sciences, University of Cape Town, National Health Laboratory Services, Cape Town, South Africa; 2 Groote Schuur Hospital, National Health Laboratory Services, Cape Town, South Africa; 3 Department of Ophthalmology, Faculty of Health Sciences, University of Cape Town, Cape Town, South Africa; 4 National Health Laboratory Services, Green Point Laboratory, Cape Town, South Africa

## Case presentation and management

A 52-year-old potato farm worker presented to his local clinic with a 1-week history of a painful left red eye. No trauma was reported, with no history of contact-lens use or eye surgery. His medical history and systemic examination were unremarkable. HIV infection and diabetes mellitus were excluded. On examination he had a large central corneal ulcer of the left eye and was referred to Groote Schuur Hospital ophthalmology for evaluation. A left eye corneal scrape was performed for microbiology and mycology, and the patient was empirically started on topical ofloxacin (one drop two hourly) and fortified cefazolin (one drop two hourly). Oral doxycycline (100 mg twice daily) and vitamin C (1000 mg twice daily) were added for the management of blepharitis with their added anti-collagenase and wound-healing properties, respectively.^1^ After 48 h of treatment, the patient developed a hypopyon with ongoing pain despite the use of analgesia. A second corneal scrape was performed with specimens sent for routine microbiology and mycology cultures. Natamycin (one drop four hourly) and gentamicin (one drop two hourly) were added topically for additional fungal and Gram-negative bacterial cover. Partial improvement of symptoms with resolution of the hypopyon was observed. Microbiological specimens, isolated *Bacillus* spp. only, prompting the cessation of antifungal therapy. Given the clinical improvement over the week, topical dexamethasone was initiated to reduce scarring and improve the patient’s visual acuity. However, with the introduction of steroids, the patient’s condition deteriorated with recurrence of the hypopyon. All treatment was stopped for 48 h and a third corneal specimen was collected for microbiology and mycology, with a suspicion that this may be fungal in nature. Topical natamycin together with ofloxacin, gentamicin and cefazolin were restarted. The third corneal specimen did not culture bacteria or fungi. Given the advanced clinical picture at this stage a glycerol-preserved corneal tissue graft was performed and the corneal specimens were sent for histology and microbiology investigations. In addition, vancomycin (one drop two hourly) to target the *Bacillus* spp. and oral ciprofloxacin (750 mg twice daily) were commenced because of limbal involvement in one quadrant from the infective process. Adnexal signs developed despite treatment and an evisceration without a ball implant was eventually performed for a progressive endophthalmitis. An urgent contrasted tomography imaging study of the brain and lumbar puncture excluded orbital and intracranial spread. Histology results showed fungal hyphae and the fourth corneal specimen cultured the mould presumptive identification as *Scedosporium* spp. resulting in the initiation of oral voriconazole (200 mg twice daily). The patient remained stable post evisceration and antifungals were continued for a total treatment duration of 3 weeks post-operatively. At follow-up visit to the ophthalmology clinic, it was observed that the wound had granulated well, with no recurrence. Subsequently, matrix-assisted laser desorption/ionisation-time of flight (MALDI-TOF) mass spectrometry (Bruker Daltonik, Germany) performed at the National Institute for Communicable Diseases (NICD), Johannesburg, South Africa confirmed the diagnosis of *S. apiospermum*.

## Important lessons learnt

1. If fungal infections are suspected deeper samples should be obtained from the stroma of the cornea. Therefore, multiple or deeper samples and proper sampling technique is required to ensure pathogen isolation.^2^
2. Antifungal susceptibility is not routinely performed on isolates of *Scedosporium* spp. Resistance to Amphotericin B and echinocandins is common, therefore voriconazole is the empiric agent for this mould. Antifungal susceptibility may be performed at a reference laboratory.^3,4^
3. Ideally all corneal samples should be referred for bacteriology and mycology investigations.
4. Maintain a high index of suspicion for fungal keratitis if vegetable or soil matter is potentially involved in the eye injury.
5. Early therapeutic corneal grafting, prior to limbal involvement, prevents scleral and systemic progression.
6. Evisceration was lifesaving in this case because disseminated infection of the involved resistant fungus is commonly fatal.

## Self-assessment questions and answers

1. What are common macroscopic and microscopic features of *Scedosporium apiospermum* shown in Figure 1?
  **Answer:** Macroscopic colonies are typically white to pale or dark grey in colour (Figure 1a). The texture is floccose with a flat to dome-shaped topography. The reverse is pale-yellow to dark-brown with age (Figure 1b). Microscopically, conidiophores are long and slender annelids that aggregate into bundles of synnemata (Graphium state). One can appreciate the characteristic ‘sweeping broom’ structure (Figure 1c, dashed box). Although not present in this picture, ascocarps (large, black, round, lemon-shaped) can be found during the sexual phase of this mould.^5^
2. With regard to pathogenesis, what are potential sources for exogenous fungal endophthalmitis?
  - Keratitis-related
  - Bleb-related
  - Device-related
  - Post-operative eye surgery
    **FIGURE 1:**
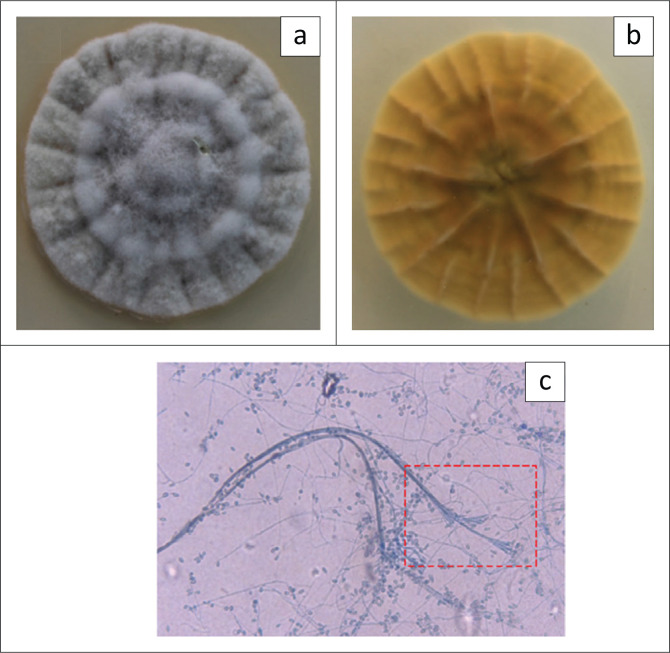
Dark-grey colonies (a) with pale-yellow reverse (b) of *Scedosporium apiospermum* macroscopically, grown at 35 °C on Sabouraud Dextrose media with amikacin. Microscopic view of *Scedosporium apiospermum* stained with lactophenol cotton blue (binocular, light microscope: 200X). The red box (dashed line) area highlights the conidiophores aggregated in the characteristic bundles of tree-like synnemata (c).
    **Answer: (a-d)** All of the given options are potential routes. Exogenous fungal endophthalmitis pathogens can be introduced from an ocular (bleb or keratitis related, post-injection, post-surgical eye procedures or device-related endophthalmitis) or environmental source (post-traumatic).^6^
3. Which of the following moulds are frequently associated with exogenous fungal endophthalmitis?
  - *Paecilomyces spp*.
  - *Fusarium spp*.
  - *Aspergillus spp*.
  - *Acremonium spp*.
    **Answer: (b, c)** All of the given moulds have been isolated in exogenous fungal endophthalmitis.^3^ *Aspergillus* and *Fusarium* spp. are common moulds implicated in exogenous endophthalmitis, whilst yeasts (*Candida* spp.) are less frequently cultured amongst fungal aetiologies in this clinical entity.^7,8^
4. Which of the following antifungal choices might be considered to treat fungal endophthalmitis?
  - Intravitreal echinocandins
  - Intravenous voriconazole
  - Intravitreal polyenes
  - Oral voriconazole
    **Answer: (a-d)** High molecular weight with low aqueous solubility antifungal drugs such as amphotericin B deoxycholate do not penetrate the blood-ocular barrier when administered parentally. However, intravitreal amphotericin B deoxycholate has a low risk for developing treatment resistance, with a broad spectrum of fungal activity and is considered relatively safe at doses of 5 µg – 10 µg in 0.1 mL. For these reasons intravitreal polyenes form the cornerstone of antifungal treatment of intraocular infections. Intravitreal caspofungin has demonstrated safety and effectiveness in the treatment of fungal endophthalmitis. Voriconazole is a triazole with good ocular bioavailability when administered intravenously or orally. Therapeutic levels are achieved in both the aqueous and vitreous humour with this antifungal.^8,9^
5. Intravitreal dosages of amphotericin B deoxycholate greater than 25 µg, commonly result in which of the following intraocular complications?
  - Cataract formation
  - Retinal necrosis
  - Glaucoma
  - Graves’ ophthalmopathy
    **Answer: (a, b)** Amphotericin B dose-related intraocular toxicity includes retinal necrosis, severe intraocular inflammation and cataract formation.^8^
